# Divergent selection in moisture‐responsive root‐branching pathways between tropical and temperate maize germplasm

**DOI:** 10.1111/jipb.70065

**Published:** 2025-11-03

**Authors:** Sunil S. Gangurde, Chenglai Wu, Jiwang Zhang, BM Prasanna, Xuecai Zhang

**Affiliations:** ^1^ CIMMYT‐China Shandong Maize and Wheat Research Center College of Agronomy, Shandong Agricultural University Tai'an 271018 China; ^2^ Borlaug Institute for South Asia (BISA) New Delhi 110012 India; ^3^ Ministry of Agriculture and Rural Affairs‐CIMMYT Maize and Wheat Joint Laboratory, Institute of Crop Sciences Chinese Academy of Agricultural Sciences (CAAS) Beijing 100081 China; ^4^ International Maize and Wheat Improvement Center (CIMMYT) Texcoco 56237 Mexico

## Abstract

This commentary on Scharwies et al. (2025, *Science*) discusses maize root branching in response to moisture gradients and highlights research gaps in investigation of the role of soil type and soil properties in driving weak or strong root hydropatterning in maize.

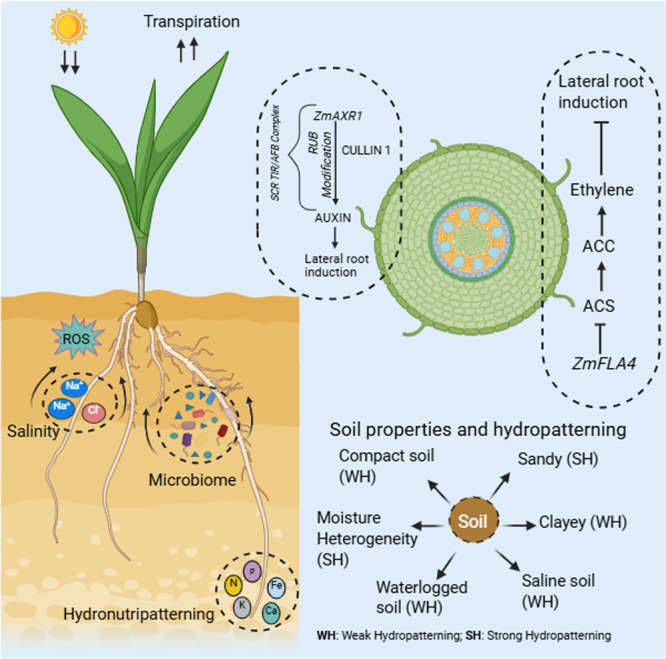

Global warming and shifting precipitation patterns are intensifying drought severity. Every 1°C rise in temperature results in ~5% soil moisture loss (Gebrechorkos et al., 2025). By 2050, it is projected that the demand for agricultural produce will be double, and 55% of global cropland will face increased drought severity risk (Christian et al., 2023). Plants absorb water via their roots. Therefore, improving root architecture will be a key for developing climate‐smart crops with enhanced drought tolerance. Trait discovery associated with roots is essential to identify the germplasms with higher root plasticity and water absorption capacity. Hydropatterning refers to the patterning of lateral roots in response to soil moisture gradients, enabling plants to enhance water uptake (Robbins and Dinneny, 2018). This phenomenon is critical for drought adaptation and nutrient acquisition. Root traits such as primary root length, lateral root density, root hairs, and mycorrhizal associations play an important role during moisture stress. Recently, Scharwies et al. (2025) developed a robust assay to study hydropatterning in maize. The results from their assay strongly agreed with previous observations in maize, rice and Arabidopsis (Robbins and Dinneny, 2018). In brief, their assay uses moist germination paper to provide moisture on one side of the primary root, called the contact‐side. The other side of the root is exposed to the drier air and referred to as the air‐side. The number of lateral roots on contact‐side and air‐side of primary roots were quantified to determine if a genotype exhibits weak or strong hydropatterning phenotypes. Plants that developed most of their lateral roots toward the moist contact‐side were called strong hydropatterning. However, the assay can be further standardized to study the effect of environmental factors such as soil moisture heterogeneity (the significant contrast between wet and dry zones), soil physical properties, soil aeration, nutrient availability. Soil texture (sand, silt, clay) and structure and porosity also affect hydropatterning. Sandy soil promotes strong hydropatterning, because large pores drain quickly and create wet and dry zones. Clayey soils hold water tightly, and this forms a uniform moisture gradient which suppresses hydropatterning. Waterlogged, anaerobic soils severely suppress root growth and hydropatterning. Soil compactness can suppress hydropatterning. Even if a zone is moist, and if the soil is too compacted, a root may be physically unable to grow toward it. Further, the roots can also grow toward a moist zone if there are available nutrients called “hydronutripatterning” (Figure 1). The authors screened a total of 250 maize inbred lines from the Goodman–Buckler association panel and found that most inbred lines generally exhibited strong hydropatterning, while fewer lines made significant quantities of air‐side lateral roots. Some species or varieties, for example drought tolerance maize Africa (DTMA) may have a more distinct hydropatterning response than others. They also tested correlations between air‐side lateral root density and field data on root crown depth and number of nodes with brace roots. However, in field conditions there are many other factors that affect root growth. For instance, clay soil has good water holding capacity but restricts root penetration. Moreover, the saline soil reduces water uptake due to osmotic stress. Soil compactness can also block the soil moisture zones, making them unapproachable to roots (Pandey and Bennett, 2024). The primary root is less plastic, therefore hydropatterning is most prominently observed in the formation of lateral roots and the elongation of root hairs. A young, developing lateral root is far more responsive to local signals than an established primary root (Figure 1). The soil microbiome critically regulates root growth through symbiosis, nutrient mobilization, and hormonal signaling. Arbuscular Mycorrhizae (AMF) can extend roots to access water and nutrients, produce strigolactones that stimulate lateral root formation, and also improve water retention around roots via glomalin‐related soil aggregates (Kakouridis et al., 2022). Certain soil microbes can alter soil water retention (e.g., through biofilm production) or produce plant hormones that influence root architecture, thereby indirectly modulating hydropatterning (Karimi et al., 2022) (Figure 1). The authors found that contact‐side lateral root density is not significantly different among tropical, sub‐tropical and temperate germplasms. However, air‐side lateral root density showed significant differences among subpopulations. Continuous selection for drought tolerance may have resulted in stronger hydropatterning in tropical and subtropical lines. With that in mind, maize diversity panels such as DTMA and water efficient maize (WEMA) that exhibit enhanced resistance to extreme soil drying is expected to have good variability for hydropatterning.

**Figure 1:**
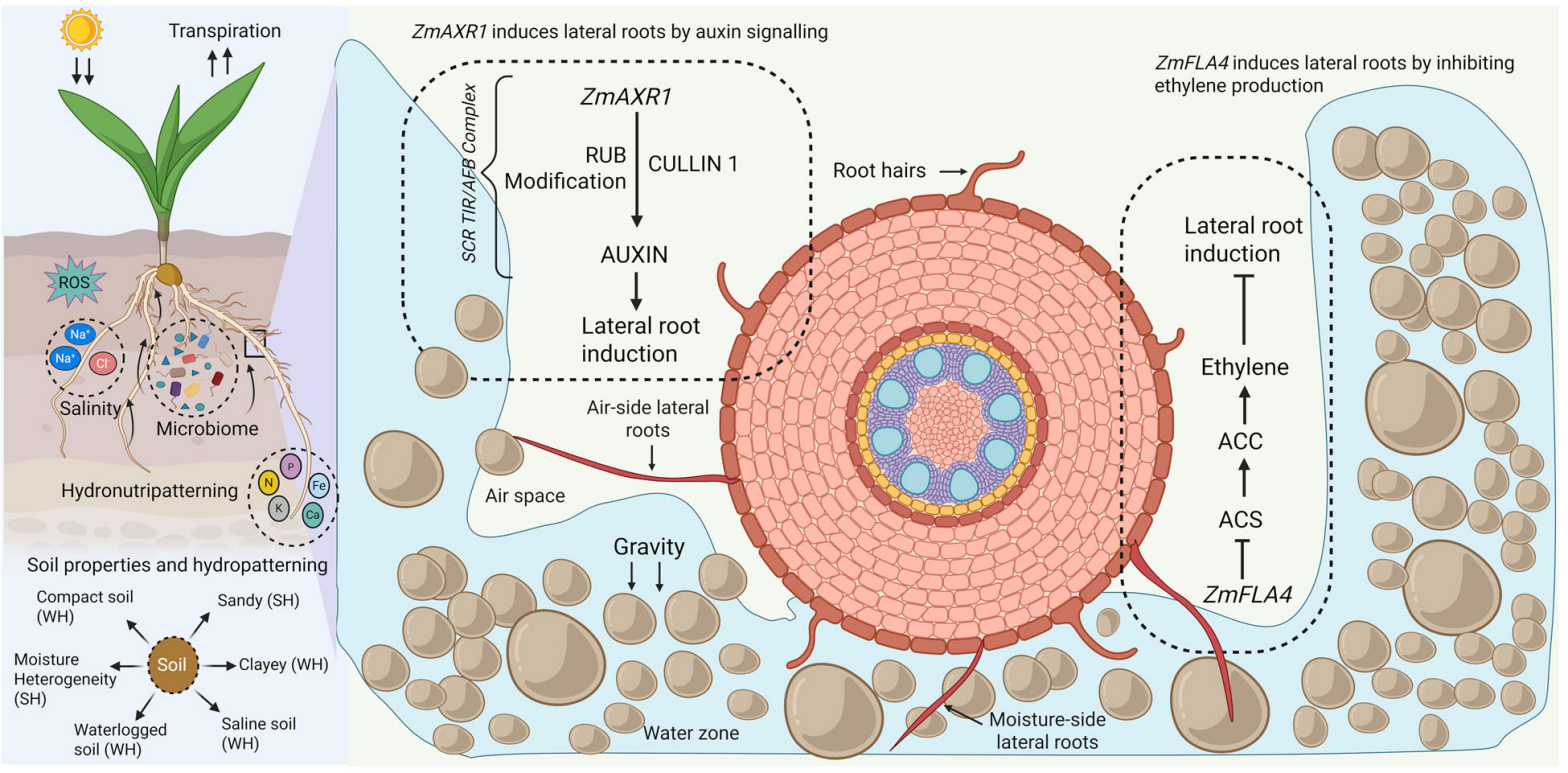
Illustration of auxin/ethylene interplay, soil properties and soil microbiome regulating the lateral root growth in maize *ZmAXR1* (*AUXIN RESISTANT1*) regulates air‐side lateral root branching by RUB (Related to Ubiquitin) modification of CULLIN 1 to produce auxin, which leads to induction of lateral root growth. The *ZmFLA4* (*FASCICLIN‐LIKE ARABINOGALACTAN PROTEIN*) inhibits ethylene production by inhibiting the activity of ACS (*AMINOCYCLOPROPANE‐1‐CARBOXILATE SYNTHASE*). There are several environmental factors that affect hydropatterning, including temperature, drought, soil salinity, soil microbiome, nutrient availability in soil. Soil types sandy or clayey soils, soil compactness, waterlogged soil and moisture heterogeneity also affect the hydropatterning in crops. In the figure, WH and SH in brackets indicate weak hydropatterning and strong hydropatterning, respectively.

## Discovery of new genetic loci associated with lateral root branching in maize

The genetic mechanisms underlying hydropatterning have been extensively characterized in *Arabidopsis thaliana*. Although the genetic network controlling this process in maize remains incompletely understood, key regulators identified in Arabidopsis—such as auxin signaling mediated by *ARF7* (*AUXIN RESPONSE FACTOR 7*) and genes involved in lateral root development like LBD (*LATERAL ORGAN BOUNDARIES‐DOMAIN*)—are likely to play conserved roles and have been implicated in related studies. During moisture stress, *ARF7* undergoes Small Ubiquitin‐like Modifier (SUMO) modification, which reduces its DNA‐binding ability and recruits inhibitory proteins like indole‐3‐acetic acid (IAA3). During availability of moisture, non‐SUMOylated *ARF7* remains active and induces lateral root initiation via *LATERAL ORGAN BOUNDARIES DOMAINS* (*LBD16*). Therefore, auxins promote lateral root growth, stimulating all stages including initiation, primordium development and emergence. In contrast cytokinin can antagonize auxin transport and signaling, suppress cell division and inhibit lateral root emergence, while role of abscisic acid is more complex; depending upon environmental conditions, it can either inhibit or permit lateral root growth. For instance, under stress it inhibits and during mild conditions it permits lateral root growth. Roy et al. (2025) recently published in *Science* that reactive oxygen species (ROS) act as a molecular switch to fine‐tune auxin signaling under water scarcity. Under drought stress, ROS induces IAA3/SHY2 multimerization via cysteine oxidation, enhancing its interaction with the co‐repressor TOPLESS (TPL) and suppressing auxin‐responsive genes to inhibit lateral root formation, this process is well known as xerobranching. Scharwies et al. (2025) uncovered through transcriptome‐wide association studies that *ZmAXR1* (*AUXIN RESISTANT1*) (*Zm00001eb211770*) is associated with air‐side lateral root density. RUB (Related to Ubiquitin) modification of CULLIN 1 (CUL1) by *ZmAXR1* produces auxin signals to induce lateral root growth on the air‐side (Figure 1). Arabidopsis *AXR1* mutants (*axr1*) exhibit shorter primary roots and reduced lateral root formation due to disrupted auxin signaling. However, in Arabidopsis, Li et al. (2015) reported that *AtAXR1* is required for ethylene‐mediated lateral root inhibition under iron (Fe) stress, linking auxin and stress responses.

Using genome‐wide association studies analysis, Scharwies et al. (2025) identified and validated *HMG‐CoA REDUCTASE DEGRADATION 3A* (*ZmHRD3A*) (*Zm00001eb398230*) and *FASCILIN‐LIKE ARABINOGALACTAN PROTEIN 4* (*ZmFLA4*) (*Zm00001eb367960*) associated with air‐side lateral root density. *HRD3A* is a key component of the endoplasmic reticulum (ER)‐associated degradation (ERAD) pathway, which degrades misfolded or unfolded proteins in the ER. It operates alongside *HRD1* and Doa10 E3 ubiquitin ligases but defines a distinct branch of ERAD in plants (Liu et al., 2024). *FLA4* plays diverse roles in plant growth, development, and stress responses, primarily related to cell wall dynamics, signaling, and stress adaptation. The minor allele of *ZmFLA4* is associated with air‐side lateral root density and lower ethylene production. Under drought stress, *ZmFLA4* may inhibit ethylene production in maize seedlings by downregulating key components of its biosynthesis pathway, including the precursor *AMINOCYCLOPROPANE‐1‐CARBOXILATE* (ACC) and the enzyme *AMINOCYCLOPROPANE‐1‐CARBOXILATE SYNTHASE* (ACS) that produces it. Under severe drought, excessive ethylene accelerates leaf senescence inhibition of lateral roots (Figure 1). Further, ethylene receptors (*ETR1*/*ERS1*) and downstream (*EIN2*/*EIN3*) transcription factors regulate stress‐responsive genes.

In future, root hydropatterning assay developed by Scharwies et al. (2025) can be customized to study the effects of soil types, soil structure and microbial inoculums to understand their role in root hydropatterning. Clustered regularly short palindromic repeats (CRISPR)/CRISPR‐associate protein 9 knockout studies for identified candidate genes could reveal whether these genes control lateral root initiation in response to moisture gradients. However, the connection between ethylene and auxin signaling in hydropatterning still needs to be investigated. Single‐cell RNA sequencing could resolve cell‐type‐specific responses to moisture gradients. Screening of diverse maize landraces (DTMA, WEMA) could identify the superior maize germplasm with strong hydropatterning for development of future drought‐smart maize.

## CONFLICTS OF INTEREST

The authors declare there are no conflicts of interest.

## AUTHOR CONTRIBUTIONS

S.S.G. and X.Z. conceptualization, S.S.G. writing ‐ original draft, review and editing, visualization, C.W. review and editing, X.Z. and J.Z. funding acquisition, project administration. All authors read and approved the final version of the manuscript.
